# Disrupting self-evaluative processing with electrostimulation mapping during awake brain surgery

**DOI:** 10.1038/s41598-021-88916-y

**Published:** 2021-04-30

**Authors:** Sam Ng, Guillaume Herbet, Anne-Laure Lemaitre, Sylvie Moritz-Gasser, Hugues Duffau

**Affiliations:** 1 Department of Neurosurgery, Gui de Chauliac Hospital, Montpellier University Medical Center, 80 Av Augustin Fliche, 34295 Montpellier, France; 2 Institut de Génomique Fonctionnelle, Université de Montpellier, CNRS, INSERM U1191, Montpellier, France; 3 Department of Speech-Language Pathology, University of Montpellier, Montpellier, France

**Keywords:** Neural circuits, Surgical oncology

## Abstract

Brain awake surgery with cognitive monitoring for tumor removal has become a standard of treatment for functional purpose. Yet, little attention has been given to patients’ interpretation and awareness of their own responses to selected cognitive tasks during direct electrostimulation (DES). We aim to report disruptions of self-evaluative processing evoked by DES during awake surgery. We further investigate cortico-subcortical structures involved in self-assessment process and report the use of an intraoperative self-assessment tool, the self-confidence index (SCI). Seventy-two patients who had undergone awake brain tumor resections were selected. Inclusion criteria were the occurrence of a DES-induced disruption of an ongoing task followed by patient’s failure to remember or criticize these impairments, or a dissociation between patient’s responses to an ongoing task and patient’s SCI. Disruptions of self-evaluation were frequently associated with semantic disorders and critical sites were mostly found along the left/right ventral semantic streams. Disconnectome analyses generated from a tractography-based atlas confirmed the high probability of the inferior fronto-occipital fasciculus to be transitory ‘disconnected’. These findings suggest that white matters pathways belonging to the ventral semantic stream may be critically involved in human self-evaluative processing. Finally, the authors discuss the implementation of the SCI task during multimodal intraoperative monitoring.

## Introduction

Awake surgery with multimodal monitoring using brain direct electrostimulation (DES) has provided oncological and functional benefits in the surgical management of diffuse low-grade gliomas (WHO grade II glioma)^1^, by allowing maximal radical excision^2^ while sparing cortical and subcortical connectivity^3,4^. In addition, neurocognitive studies based on intraoperative on-line responses to DES have made possible to better understand and preserve neural pathways subserving motor and language functions, but also higher order cognitive functions such as visuospatial cognition^5^, mentalizing^6,7^ and semantic cognition^8,9^. Yet, little attention has been given to patients’ interpretation of their own responses during electrostimulation. Previous reports demonstrated that DES of the ventral semantic stream may induce not only errors during naming tasks or semantic association tasks, but also complex subjective experiences, including transient disruptions of patients’ normal state of consciousness^10^.

It has been recently reported that patients may still suffer from mild neurocognitive disturbances after surgery despite awake monitoring (including long-lasting executive functioning impairment^11^, decrease of attentional processes^12^ or behavioral changes^13,14^). This prompted our group to ask patients if they were aware of their DES-induced disorders while performing intraoperative tasks.

In this brain electrostimulation mapping study, first, we collected all consecutive cases of DES-generated disruption of self-evaluation in order to identify critical cortico-subcortical structures that might be involved. Second, we detailed two clinical illustrative cases of DES-generated disruption of self-evaluative processing during awake surgery. At last, we discuss potential implications of these findings for DES-guided brain tumor resections, especially by proposing the addition of a self-confidence task during awake procedures.

## Results

### Participants

We selected 72 patients according to the inclusion criteria (36 women and 36 men), with a mean age of 40.9 ± 10.8 years (range 20–63 years). Fifty-three were right-handed, 14 were left-handed, and 5 were ambidextrous. Seventy-four electrostimulations sites were eligible (2 patients presented self-evaluation disorders in 2 distinct sites of electrostimulation). Tumors were located in the frontal lobe in 18 patients, in the temporal lobe in 18 patients, in the parietal lobe in 5 patients, in the insular lobe in 5 patients and in multiple lobes, including the insula in 26 patients. Preoperative cognitive data are provided by tasks and cognitive domains (i.e. language, psychomotor speed, executive functioning, episodic memory, visuospatial functioning, social cognition) in Table 1. Note that neuropsychological scores are expressed as Z-scores based on French published normative data. The mean preoperative tumor volume was 50.7 ± 45.1 cm^3^ (range 2–169 cm^3^) and the mean postoperative tumoral residue (measured on the 3-month postoperative MRI) was 4.9 ± 5.7 cm^3^ (range 0–24 cm^3^).

**Table 1:** Presurgical neuropsychological test results.

Test	Mean Z-score (SD)
**Language and semantics**	
DO80^a^	0.26 (0.63)
PPTT^a^	0.06 (1.41)
Phonological fluency	− 0.19 (1.32)
Categorical fluency	− 0.28 (1.33)
Reading text^a^	− 0.42 (0.88)
Regular words^a^	0.03 (0.87)
Irregular words^a^	0.51 (0.67)
Pseudo-words^a^	0.23 (1.12)
**Executive functioning**	
TMT B	0.19 (1.01)
TMT B-A	− 0.18 (1.15)
STROOP interference	0.20 (1.37)
STROOP I-D	0.04 (1.04)
Phonological fluency	− 0.19 (1.32)
Categorical fluency	− 0.28 (1.33)
Forward span	− 0.11 (1.26)
Backward span	− 0.19 (0.94)
**Psychomotor speed and attention**	
WAIS IV Code	0.36 (0.87)
TMT A	0.53 (0.70)
STROOP naming	0.22 (1.35)
STROOP reading	0.19 (1.47)
**Episodic memory**	
RL-RI16: 1st free recall	− 0.30 (0.44)
RL-RI16: 2nd free recall	− 0.45 (1.01)
RL-RI16: 3rd free recall	− 0.28 (1.06)
RL-RI16: delayed recall	− 0.46 (0.96)
**Visuospatial functioning**	
ROCF: copy^b^	0.97 (0.33)
ROCF: immediate recall^b^	0.25 (0.84)
ROCF: delayed recall^b^	0.37 (0.74)
TCF: copy^b^	0.73 (0.42)
TCF: immediate recall^b^	0.27 (1.44)
TCF: delayed recall^b^	− 0.29 (1.42)
Bell test	0.47 (0.87)
**Social cognition**	
RME	− 0.11 (1.06)

*DO80* Dénomination orale à 80 items, *PPTT* Pyramid and palm tree test, *TMT* trail making test, *RL-RI16* Rappel libre rappel indicé, *ROCF* Rey–Osterrieth Complex Figure, *RME* read the mind in the eyes task, *TCF* Taylor Complex Figure, *WAIS IV* Wechsler adult intelligence scale IV.

Data were available for 55 patients in language and executive functioning domain, for 48 patients in psychomotor speed and attention domain, for 40 patients in episodic memory domain, for 48 patients in visuospatial functioning domain and 28 patients in social cognition domain. Patients without presurgical RME evaluation were not evaluated with the RME task intraoperatively.

^a^Some language tasks were assessed only for patients with left-sided tumors and/or left-handed patients.

^b^Rey-Osterrieth and Taylor complex figures were randomly switched between the presurgical and postsurgical examination.

### Intraoperative findings

During surgery, cortical and subcortical mapping were achieved while patients performed cognitive tasks (e.g. picture naming, the Pyramids and Palm Trees Test [PPTT], the Read the Mind in the Eyes [RME] test and double tasks) continuously. The average current intensity was 2.32 ± 0.5 mA. The self confidence index (SCI) was combined with the RME task and systematically administered preoperatively. In this way, items that resulted in failed responses at the RME task were excluded from the intraoperative materials, thus reducing the likelihood of false positive responses and making the task easier for the intraoperative assessment. Overall, 74 stimulation sites were identified: 8 of them were cortical stimulations and 66 of them were subcortical stimulations. Within the left hemisphere (46 electrostimulation sites), self-evaluative disorders were associated to semantic disorders in 45/46 patients, with a frequent association with semantic paraphasia or anomia (35/46) and non-verbal semantic-association disorders (8/46, reported as a failure to complete a PPTT trial). Most of the time, left stimulations induced massive difficulties to remember and criticize the episode of semantic loss (with the notable exception of the second illustrative case, see below). Within the right hemisphere (28 electrostimulation sites), self-evaluation disturbances were mostly detected with the RME/SCI task (15/28). Note that in all cases, the onset of RME/SCI dissociations leaded the surgeon to stop the resection.

### Cortical and subcortical spatial distribution

**Figure 1:**
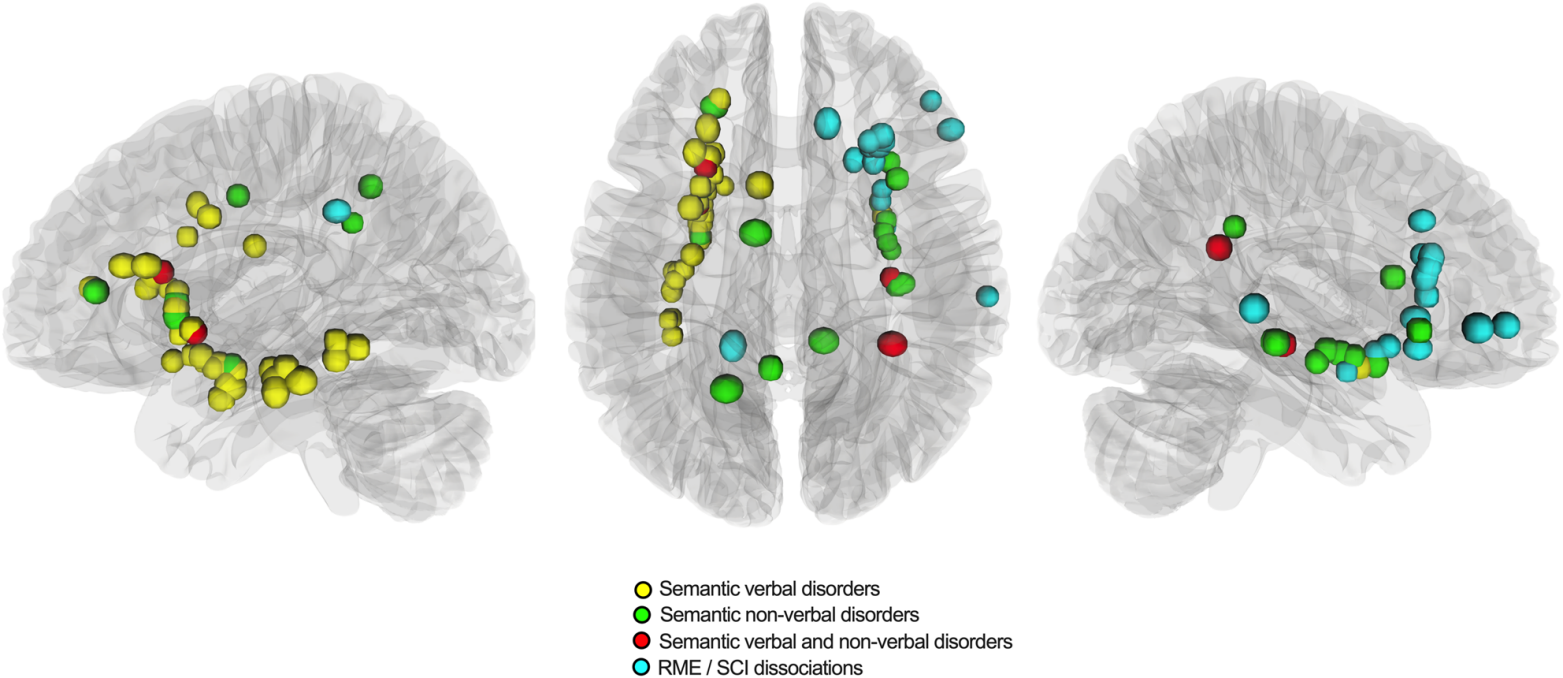
Distribution of cortical and subcortical sites generating self-evaluative disorders. The left part of the figure describes the sites from the left hemisphere (n = 46), the right part of the figure describes the sites from the right hemisphere (n = 28) and the central part of the figure describes all stimulation points, from a superior axial view (n = 74). Yellow circles indicate associated verbal semantic disorders, green circles indicate associated non-verbal semantic disorders, red circles indicate both verbal and non-verbal semantic disorders and cyan circles indicate dissociations between Read the Mind in the Eyes task and the self-confidence index task.

The spatial topography of cortical and subcortical sites eliciting self-evaluation impairments is provided in Fig. 1. Overall, 38 sites (35 in left hemisphere, 3 in right hemisphere) induced verbal semantic disorders and a disruption of self-evaluative processing (*yellow circles*), 16 sites (8 in left hemisphere, 8 in right hemisphere) induced non-verbal semantic disorders and a disruption of self-evaluative processing (*green circles*), 4 sites (2 in left hemisphere, 2 in right hemisphere) induced both verbal and non-verbal semantic disorders and a disruption of self-evaluative processing (*red circles*) and 16 sites (1 in left hemisphere, 15 in right hemisphere) elicited isolated dissociations during the RME/SCI task (*cyan circles*).

### Neural pathways disconnected during DES

Individual maximal probabilities of disconnection of the main white matter tracts and related MNI coordinates are displayed in Supplementary information—Results. Average p_max_ within the inferior fronto-occipital fasciculus (IFOF), the inferior longitudinal fasciculus (ILF), the uncinate fasciculus (UF), the middle longitudinal fasciculus (MdLF), the superior longitudinal fasciculus (SLF), the arcuate fasciculus (AF) and the cingulum are detailed in Table 2.

**Table 2:** Results of probabilistic disconnectome maps and subsequent disconnected white matter pathways in direct electrostimulations eliciting impairments of self-evaluative processing (N = 74).

White matter pathways^a^	All stimulations (N = 74)			Left fronto-temporo-insular stimulations (N = 41)		Right fronto-temporo-insular stimulations (N = 25)			Left and Right parietal and cingulum stimulations (N = 8)	
	Average P_max_	95% CI		Average P_max_	95% CI	Average P_max_	95% CI		Average P_max_	95% CI
**IFOF**^b^	0.77	0.69–0.85		0.91	0.86–0.95	0.74	0.59–0.89		0.16	0.00–0.42
Frontal component	0.6	0.52–0.68		0.44	0.32–0.57	0.60	0.45–0.74		0.10	0.00–0.26
Insular component	0.72	0.64–0.81		0.83	0.76–0.91	0.71	0.55–0.88		0.16	0.00–0.42
Occipital component	0.61	0.52–0.69		0.69	0.60–0.77	0.64	0.48–0.80		0.07	0.00–0.25
**ILF**^b^	0.63	0.55–0.70		0.77	0.72–0.82	0.57	0.42–0.72		0.05	0.00–0.18
Temporal component	0.45	0.37–0.54		0.58	0.48–0.67	0.39	0.23–0.56		0.02	0.00–0.06
Occipital component	0.56	0.48–0.63		0.67	0.59–0.75	0.53	0.40–0.67		0.05	0.00–0.18
**UF**^b^	0.39	0.29–0.48		0.48	0.35–0.61	0.36	0.19–0.52		0.00	0.00–0.00
Frontal component	0.35	0.26–0.44		0.45	0.32–0.57	0.30	0.14–0.46		0.00	0.00–0.00
Temporal component	0.29	0.21–0.38		0.33	0.21–0.45	0.32	0.16–0.47		0.00	0.00–0.00
**MdLF**^b^	0.16	0.11–0.20		0.15	0.09–0.21	0.17	0.10–0.24		0.15	0.00–0.37
Parietal component	0.13	0.09–0.17		0.12	0.07–0.18	0.13	0.07–0.20		0.14	0.00–0.37
Temporal component	0.10	0.07–0.14		0.09	0.04–0.14	0.13	0.07–0.19		0.08	0.00–0.22
**SLF**^b^	0.27	0.21–0.33		0.30	0.21–0.39	0.29	0.20–0.37		0.03	0.00–0.09
Frontal component	0.26	0.20–0.32		0.29	0.20–0.38	0.29	0.20–0.37		0.03	0.00–0.09
Parietal component	0.04	0.02–0.06		0.05	0.02–0.08	0.05	0.02–0.07		0.00	0.00–0.00
**Arcuate**^b^	0.36	0.29–0.43		0.36	0.26–0.46	0.46	0.34–0.58		0.08	0.00–0.28
Frontal component	0.26	0.19–0.32		0.26	0.16–0.36	0.31	0.20–0.41		0.06	0.00–0.20
Temporal component	0.27	0.21–0.34		0.28	0.19–0.36	0.33	0.22–0.44		0.08	0.00–0.28
Cingulum	0.07	0.02–0.11		0.01	0.00–0.02	0.01	0.00–0.02		0.54	0.22–0.85

*CI* confidence interval, *IFOF* inferior fronto-occipital fasciculus, *ILF* inferior longitudinal fasciculus, *MdLF* middle longitudinal fasciculus, *SLF* superior longitudinal fasciculus, *UF* uncinate fasciculus.

^a^Individual disconnectome maps were confronted to the Human Connectome Project 842 tractography database. Additional information concerning white matter tracts segmentation are provided in Supplemental data 1.

^b^Average P_max_ for each white matter tract corresponds to the average of each maximal probability of disconnection for this tract, including all its subcomponents.

**Figure 2:**
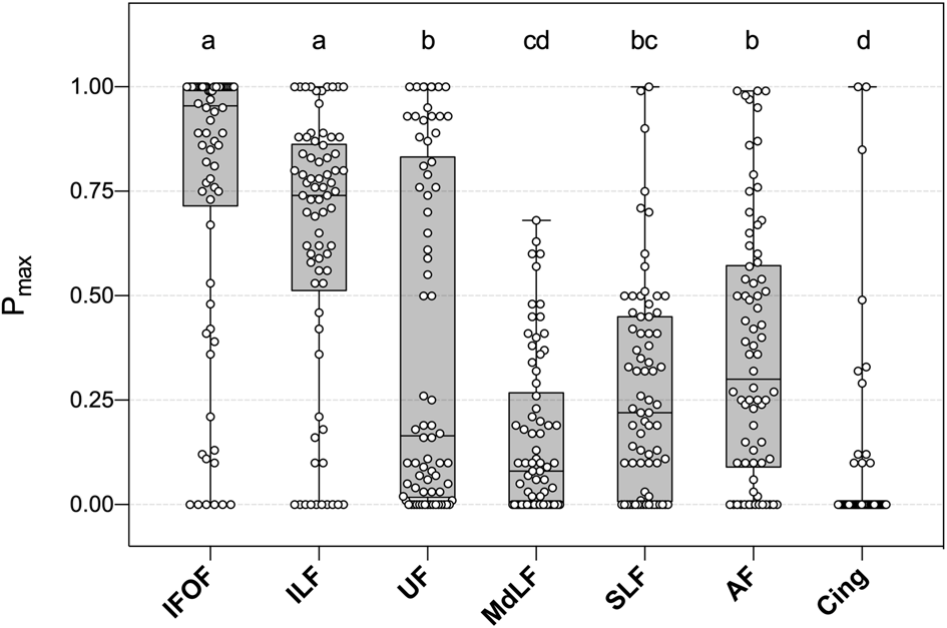
Box plots reporting the values of p_max_ within the main white matter tracts in 74 electrostimulations. *AF* arcuate fasciculus, *Cing* cingulum, *ILF* inferior longitudinal fasciculus, *IFOF* inferior fronto-occipital tract, *MdLF* middle longitudinal fasciculus, *SLF* superior longitudinal fasciculus, *UF* Uncinate fasciculus. p_max_is the maximal probability of disconnection for a given white matter tract. Data are presented as median, quartiles and extreme values. Different letters indicate statistical significance with an adjusted P-value < .05.

Overall, the values of p_max_ were significantly different within the main white matter tracts (H = 190.6, P < 0.0001, d.f. = 6, Fig. 2). The IFOF and the ILF were associated with higher values of p_max_ in comparison with the UF (P < 0.0001 and P = 0.02, respectively), the AF (P < 0.0001 and P = 0.01, respectively), the MdLF (P < 0.0001 in both comparisons), the SLF (P < 0.0001 in both comparisons), and the cingulum (P < 0.0001 in both comparisons). There was no statistical difference between the IFOF and the ILF (P = 0.48).

**Figure 3:**
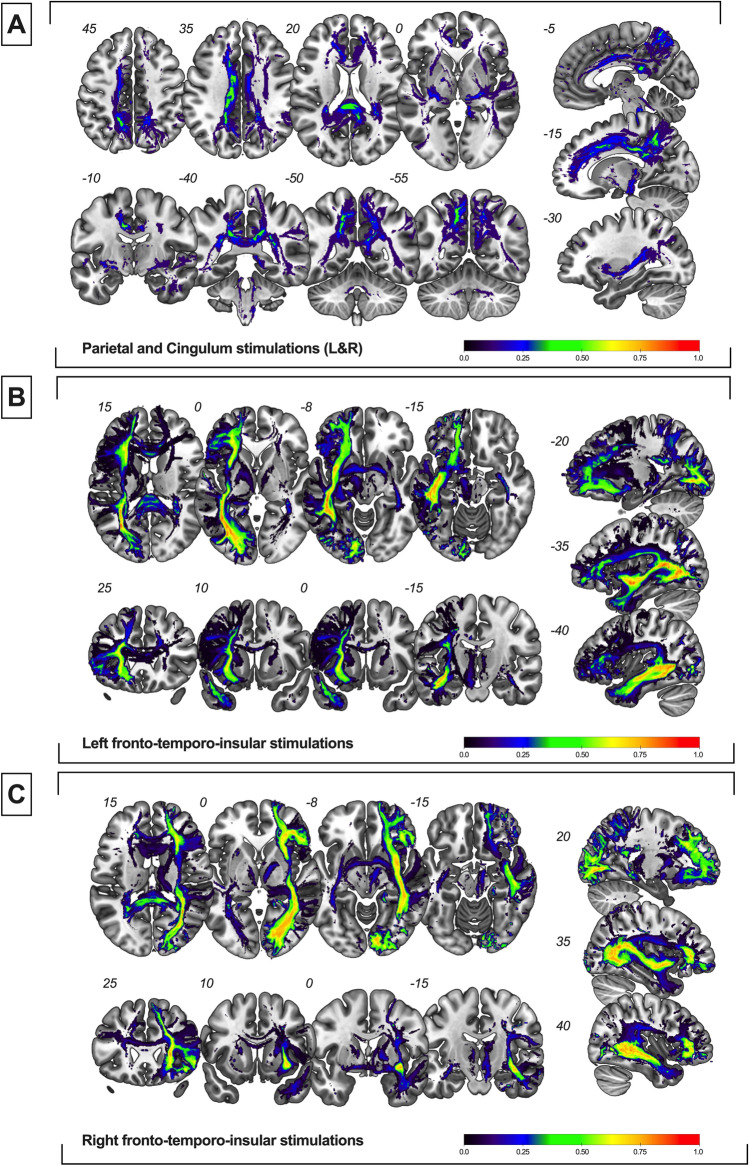
Average disconnectome maps for disruptions of self-evaluative processing. **(A**) Average disconnectome maps generated with bilateral posteromedial sites (n = 8). (**B**) Average disconnectome maps generated with left fronto-temporo-insular sites (n = 41). (**C**) Average disconnectome maps generated with right fronto-temporo-insular sites (n = 25).

Average disconnectome maps showing the overlapping of white matters tracts “disconnected” during DES-induced self-evaluation impairments are provided in Fig. 3.

### Illustrative cases

#### Case 1

**Figure 4:**
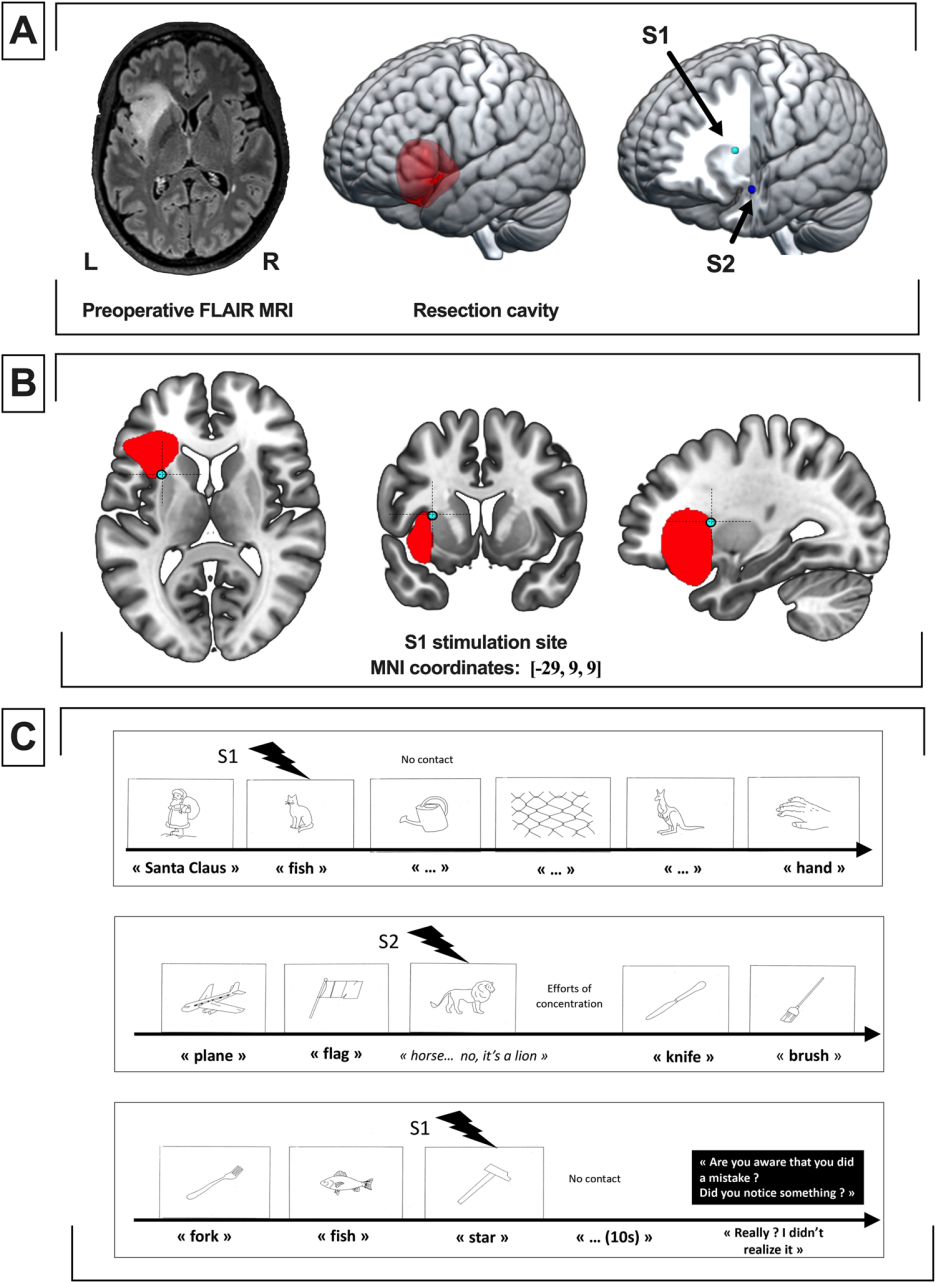
Case illustration 1. S1: Illustrative stimulation 1, generating verbal semantic disorders and a disruption of self-evaluative processing. S2: Illustrative stimulation 2, generating verbal semantic disorders without self-evaluative disturbances. (**A**) Preoperative FLAIR MRI axial sequence (left), 3D rendering of the resection cavity (center) and 3D rendering of the stimulation points S1 and S2. (**B**) Axial (left), coronal (center) and sagittal (right) location of S1 stimulation site. The cavity of resection is presented in red. (**C**) Schematic description of the intraoperative protocol (DO80 naming task).

This 38-year-old right-handed woman with a previous history of hypothyroidism, sales agent, had an incidental discovery of a left fronto-insular lesion highly suggestive of low-grade glioma measuring 30 cm^3^ (non-enhancing hypointense lesion on T1-weighted MRI, hyperintense lesion on FLAIR MRI, Fig. 4A). Neuropsychological examination demonstrated no cognitive impairment. Due to volumetric expansion, surgery was proposed under awake condition to perform intraoperative multimodal mapping. Standard procedure was achieved to expose the left fronto-temporal region. The electrical stimulation was set at 60 Hz, 2.5 mA. A subpial dissection through the pars triangularis and the pars orbitalis was performed to reach the left insular lobe and remove the tumor.

Two functional sites were identified at the subcortical level (see Fig. 4A,B). A schematic description of the intraoperative testing protocol is provided in Fig. 4C.

Stimulation of the upper portion of the insula located under the superior limiting sulcus (S1) elicited a semantic paraphasia (the patient answered “fish” instead of “cat”) and an absence of response during 3 other items (≈12 s). At this time, the patient was unable to point out anything, with a facial expression of incomprehension. Stimulation located near the inferior limiting sulcus of the insula (S2) elicited semantic paraphasia with intense attempts of self-correction (the patient told “horse” then hesitated and finally found the right item “lion” spontaneously in a few second, suggesting that self-evaluative processes were not disrupted during this stimulation).

Later, a second stimulation at the S1 location was performed: the patient answered “star” instead of “hammer”, then stopped to produce responses. When she recovered all normal language abilities and a normal state of “consciousness”, she was asked if she was aware of her troubles and what she felt during the stimulation. She answered with surprise that she was strictly not aware of any troubles and that she did not remember any particular event.

#### Case 2

**Figure 5:**
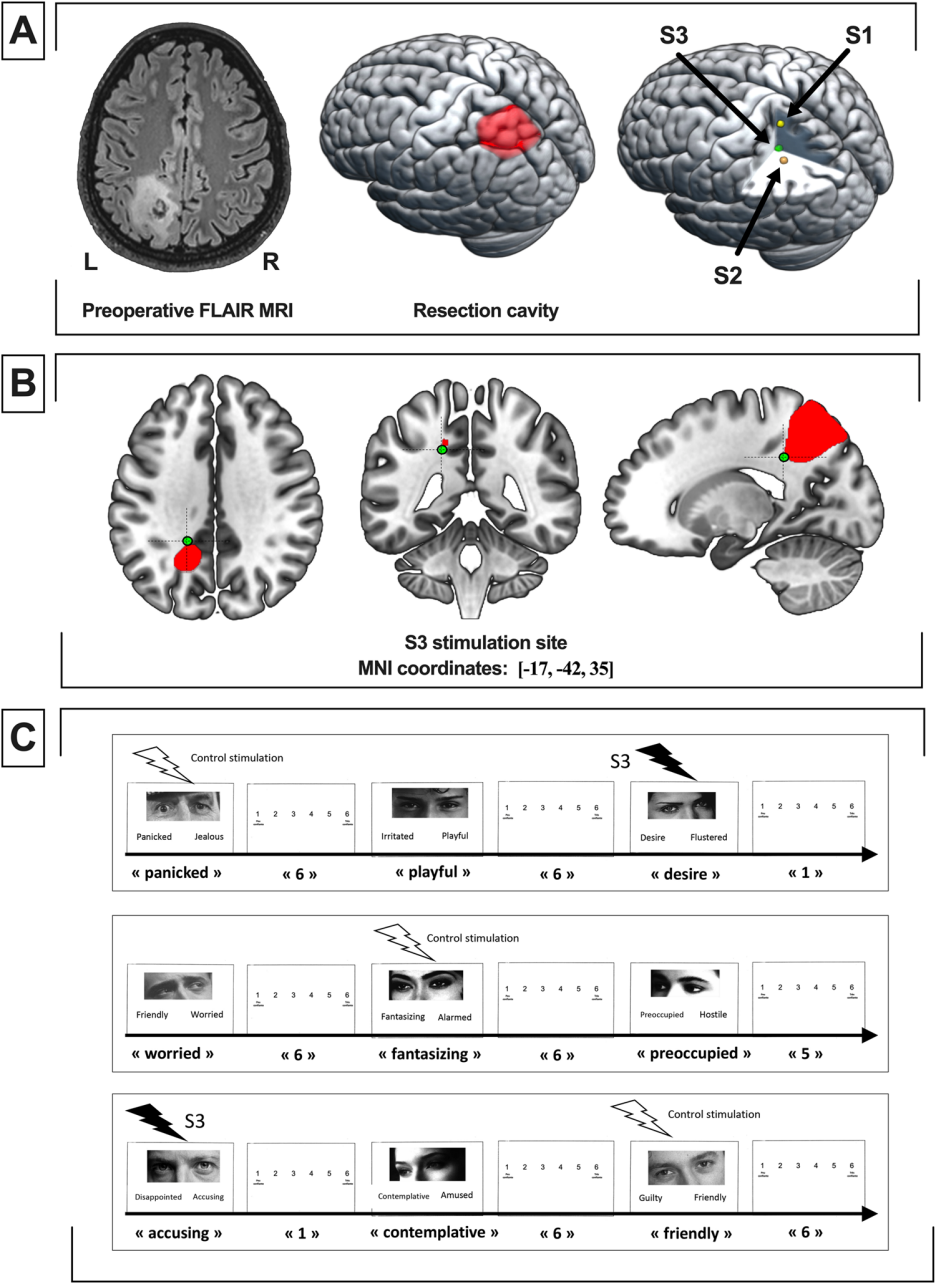
Case illustration 2. S1: Illustrative stimulation 1, generating right leg dysesthesias and thrilling. S2: Illustrative stimulation 2, generating anomia. S3: Illustrative stimulation 3, generating a dissociation between the Read the mind in the Eyes task and the self-confidence index task. (**A**) Preoperative FLAIR MRI axial sequence (left), 3D rendering of the resection cavity (center) and 3D rendering of the stimulation points S1, S2 and S3. (**B**) Axial (left), coronal (center) and sagittal (right) location of S1 stimulation site. The cavity of resection is presented in red. (**C**) Schematic description of the intraoperative protocol (read the mind in the Eyes task and self-confidence index task).

This 39-year-old left-handed woman without previous medical history, nurse, had an incidental discovery of a left superior parietal lobule lesion highly suggestive of low-grade glioma measuring 53 cm^3^ (non-enhancing hypointense lesion on T1-weighted MRI, hyperintense lesion on FLAIR MRI, Fig. 5A). The patient reported subjective complaints of working memory disorders, but neuropsychological examination demonstrated no cognitive impairment. Due to volumetric expansion, a surgical resection under awake condition was proposed. Intraoperative electrical settings were 60 Hz and 2.0 mA. Briefly, cortical stimulations of the retro-central and paracentral lobules elicited hand and upper limb dysesthesias, respectively. Subcortical responses to DES are presented in Fig. 5A. Anteriorly, right leg dysesthesias and trilling were induced by the stimulation S1 (thalamo-cortical fibers). Posteriorly and laterally, anomia was induced by the stimulation S2 (arcuate fasciculus).

Stimulations S3, located near the posterior cingulum (see Fig. 5B) generated no errors to the RME task, but systematic and reversible decrease of the SCI. A schematic description of the intraoperative protocol is provided in Fig. 5C.

## Discussion

Given the oncological impact of maximal resection in low-grade gliomas^15^, awake mapping techniques have been significantly developed in neurosurgical centers. Nevertheless, since surgical management is now proposed earlier for oncological purpose (even in asymptomatic patients with incidental low-grade gliomas)^16^, expectations must be set higher in terms of functional outcomes in order to preserve health-related quality of life, including return to work. For this purpose, the reliability of brain mapping has been gradually implemented by intraoperative tasks over the last two decades. For example, it was demonstrated that the PPTT allowed a better monitoring of non-verbal semantic outputs during awake surgery^10^. In the same vein, it was reported that resections of the right posterior inferior frontal gyrus leaded to deficits of low-level mentalizing processes^17^, thus justifying the use of the RME task to monitor more efficiently neural pathways subserving face-based mentalizing^7^.

Although DES mapping is now widely acknowledged as an effective means of accurately mapping domain-specific functions, only few reports have established its potential for identifying the neural circuitry involved in complex, higher-order cognitions^18^. In this study, the advantage of DES mapping over other imaging modalities lies in its ability to gauge in real-time the causal implication of long-range connective tracts in self-evaluation abilities, by preventing remote associative, multimodal areas to communicate (i.e. transient disconnection). As self-evaluative processing is likely to rely on the resources of multiple functional networks (e.g. memory or attention among others), the main finding of this study does not rule out the possibility that other structures may participate in this complex function.

Some authors—either by the mean of stereo-encephalographic cortical stimulations^19^ or using awake mapping during brain tumor resections^20^—have reported complex subjective experiences like behavioral unresponsiveness or loss of connectedness, that were often classified as psychic phenomenon. Other studies have suggested that similar manifestations may occur during multimodal semantic impairments generated by electrostimulation^10^ (namely, an “absence of response” followed by an incapacity to remember the “episode of semantic loss”). Our results support that DES of the ventral semantic stream (underpinned mainly by the IFOF) and of the posteromedial structures may disrupt self-evaluative processing. As the IFOF/ILF fascicles have been reportedly associated with semantic cognition, we suggest that a transient self-evaluative disability may be related to a more complex and amodal disorganization of semantic cognition^10^ and, speculatively, to a disturbance of self-directed cognition, reinforcing the idea that there is a link between these two aspects of cognition^21^. This assumption is additionally supported by the fact that multimodal disruptions of semantic processes were generated during DES. Indeed, patients frequently had verbal semantic disorders (see case illustration, Fig. 3), non-verbal semantic disorders, and a breakdown in their memory processing since most of them declared difficulties to remember their own answers. Interestingly, both self-evaluation and verbal semantic or non-verbal semantic outputs were transiently disturbed at the same time, suggesting a disruption of the feedback mechanism relevant to lexical-semantic encoding. This is in line with the comprehension-based model of lexical access by Levelt and colleagues (WAIVER++)^22^ which introduced self-monitoring as an integral part of speech planning and production^23^ during inner as well as overt speech processing.

Several studies have described the microstructure of the IFOF, an associative white matter pathway composed of multiple components connecting the frontal, the temporal, the parietal and the occipital lobes^24^, with up to five different layers^25^. The functional roles of this fascicle are mostly unknown to date, but implications have been suggested in semantic processing^26^, executive control of voluntary visual recall^27^ and conscious visual processing^28^. It is worth mentioning that reported stimulations of the right hemisphere elicited subtle changes of self-evaluative processing: patients had generally a preservation of their “normal state of consciousness” and self-evaluative impairment could only be detected with the RME/SCI tasks. By contrast, left stimulations of the IFOF generally elicited interruptions of apparent consciousness. These observations are consistent with previous studies reporting that the left IFOF might be more markedly involved in semantic control than the right one^29^.

Another speculative assumption is that self-evaluative disturbances reported in this study might consist in an inter-system breakdown, triggered by a critical “disconnection” of the ventral semantic stream, underpinned by the IFOF and/or the ILF. This so-called “third level of neural disruption” was originally described to explain DES-induced disturbances of multiple function-specific networks leading to complex behavioral disturbances (e.g. the inability to perform multiple task simultaneously)^30^. The presence of a small cluster of functional sites distant to the ventral semantic stream (notably located within the posterior cingulate cortex) might support this theoretical model. Indeed, anatomical and neuroimaging studies have reported the pivotal role of the posterior cingulate cortex, as a potential “connector hub” involved not only in the default mode network, but also in large-scale networks including numerous interconnections with distant areas of the cerebral hemispheres^31^. In addition, this speculative hypothesis is further corroborated by a recent structural study showing that the IFOF, the cingulum and the corpus callosum play a critical role in conscious access in psychotic patients^32^, thus supporting the idea that long-distance connectivity allows a global neuronal ignition coding for conscious content^33^.

Finally, the authors are currently discussing the implementation of multimodal intraoperative evaluations combined with the self-confidence index task (e.g. PPTT/SCI task, DO/SCI task in addition to the RME/SCI task) to better monitor self-evaluative processing during awake surgery and investigate its modal/amodal specificity. This task may be easily integrated to current intraoperative evaluations with a minimum amount of additional time (4 s per item) and without increasing the risk of intraoperative seizure because it does not require further DES. In addition, this task may increase the sensitivity of current routine tasks such as semantic association tasks. Of note, complete clinical and neuropsychological investigations will definitely be required to demonstrate the validity of this test and its potential clinical benefits^34^.

### Limitations

It could be argued that the observations described in this study are partly based on subjective reports of the patients. However, similar subjective descriptions have previously contributed in grasping the neurocognitive mechanisms underlying complex cognitive phenomena, especially in stimulation studies (e.g. movement intention^35^, sensory experiences^36^ or consciousness from the external environment^20^). Furthermore, authors’ proposal to implement the SCI task could be a useful intraoperative tool to objectify these clinical observations. Another limitation is that minor self-evaluation disorders may have been underdiagnosed, especially in the left hemisphere. Indeed, the SCI task was specifically combined to the RME task (mostly used in the right hemisphere). In addition, the authors acknowledge that interferences with mentalizing disturbances may have occurred when RME/SCI dissociations were reported, thus raising the question of the phenomenological continuity between such dissociations and more massive disruptions of self-evaluative processing. The addition of the SCI task combined with other intraoperative tasks could definitely represent a helpful tool to investigate the modal-specificity of this phenomenon.

Furthermore, although the tumor volume could be associated with substantial mass effect in few patients, we used the 3-month postoperative MRI to spatially position electrostimulation sites. Also, note that the mean postoperative volume was 4.9 ± 5.7 cm^3^, thus limiting possible biases related to mass effect. In addition, it is known that tumor diffusion along the surrounding white matter fibers exposes to a significant likelihood of false-negative and/or false-positive fibers, depending on the DTI processing method used. This is the reason why we decided to use white matter tractography atlases to estimate and identify which white matter fibers were ‘disconnected’ by subcortical electrostimulations.

Finally, intraoperative electro-encephalography recordings were not performed systematically. The occurrence of afterdischarges was not evaluated. However, we previously showed that our intraoperative protocol is associated with a very limited rate of intraoperative seizure without using intraoperative electro-corticography monitoring^37^ (3.4% over 374 patients, with a mean current intensity of 2.25 ± 0.6 mA while the average current intensity in the present study was 2.32 ± 0.5 mA). Also, most positive sites were related to white matter electrostimulations (66/74), which drastically reduce the likelihood of afterdischarges.

## Conclusion

In this observational brain electrostimulation study based on intraoperative findings in 72 awake surgical procedures, we report that disruptions of self-evaluative processing may be evoked by DES. Our findings suggest that the ventral semantic stream (mainly underpinned by the IFOF and the ILF), and to some extent that posteromedial structures may be involved in self-evaluative processing. Therefore, the authors suggest the implementation of a self-confidence index task during multimodal intraoperative monitoring, although its feasibility and validity remains to be confirmed.

## Methods

### Study design

A retrospective analysis of a consecutive series was conducted. Screened patients had undergone surgical resection of a diffuse glioma under awake condition in a single center between January 2013 and January 2020. All patients were operated on by the same neurosurgeon (H.D). It should be noted that the data presented in this study were gained in a clinical context, and that the protocol described below reflects our standard clinical approach. Approval for the study and its methods was granted by the Institutional Review Board of Montpellier University Medical Center (N°202000557). All patients gave their informed consent prior to inclusion. All methods of this study were performed in compliance with the tenets of the Declaration of Helsinki. Data and imaging were studied following anonymization in agreement with the Personal Data Protection Act and the code of conduct for responsible use of Human Tissue and Medical Research.

### Surgical approach

The surgical procedure of cortical and subcortical electrical mapping in patients undergoing asleep-awake-asleep craniotomy has been exhaustively reported elsewhere^3^. After craniotomy and dural opening, the cortical surface was exposed, and the edges of the tumor were visualized by intraoperative ultrasound and indicated with sterile letter tags. Once the patient was awake, electrical mapping was performed with a bipolar electrode probe with 5 mm inter-tip spacing (NIMBUS Stimulator, Newmedic, France), delivering a biphasic electric current (60 Hz, 1 ms pulse width, amplitude 1.5–4 mA). The amplitude was gradually increased until a positive response from the ventral premotor cortex was obtained (i.e. transient speech articulatory disturbances). This amplitude was not modified during the remainder of the intraoperative mapping (including both cortical and subcortical sequences). In order to restrain the spread of the electric current to the adjacent brain structures, the duration of DES never exceeded 4 s. Intraoperative assessment of cognitive functions was achieved by a dedicated speech therapist (SMG) and/or neuropsychologist (GH/ A-LL), blinded to the stimulation application. After completion of the cortical mapping, subpial dissection and tumor removal was started, allowing an access to the white matter pathways. Subcortical DES mapping was performed to achieve tumor resection according to individual functional boundaries. Two photographs were taken: one at the end of the cortical mapping and one at the end of the resection.

### Intraoperative cognitive tasks

Depending on the tumor site, multimodal cognitive mapping was performed during the awake procedure, including a motor task, a language naming task “DO80”^38^, a semantic association task (Pyramid and palm-tree test, PPTT)^39^, a line bisection task, dual-tasks (picture plus movement)^40^, a reading task and an mentalizing task (Read the Mind in the Eyes task, RME)^41^. An index reflecting the level of confidence of the patient in his own response (ranging from 1 to 6 gradually reflecting the level of confidence of the patient, 6 being the most confident response) was combined to the RME task. This self-confidence index (SCI) task was originally described as a subcomponent of the RME task in order to increase its sensitivity^42^.

Other intra-operative disturbances were recorded as follows: (1) inabilities to name visually presented pictures were accounted as anomias; (2) incorrect but semantically related picture naming (e.g. “mouse” instead of “cat”) were accounted as verbal semantic disorders and (3) failures to complete a PPTT trial were accounted as non-verbal semantic disorders.

Note that all patients were tested with the above-mentioned tasks the day before the surgery. In this way, failed items during this preoperative examination were excluded from the intraoperative materials, thus reducing the likelihood of false positive responses. Moreover, the RME task was adapted for the surgical procedure in the same way that described in Herbet et al.^43^ and Yordanova et al.^7^ In particular, only two items were used instead of 4 in the original version of the task. This allowed to drastically diminish the rate of false positive responses due to task difficulty and to maintain SCI at a high rate. Using this method, all selected items were associated with a SCI > 4/6.

### Patients selection

Inclusion criteria were the occurrence of at least one of the following reproducible and reversible DES-generated event(s), assessed by a dedicated neuropsychologist and/or speech therapist: (1) a clinical disruption of self-evaluative processing, defined by an error or an absence of response to an ongoing task (namely, during a naming task [DO80] or semantic association task [PPTT]), followed by patient’s disability to remember or criticize these phenomena after the end of the DES (once the patient had completely recovered his language abilities and his normal state of consciousness); (2) a reproducible dissociation between patient’s responses to the RME^6,7^ and patient’s responses to the SCI task (ranging from 1 to 6 gradually reflecting the level of confidence of the patient in his own responses). Such dissociations at the RME/SCI tasks occurred when patients produced correct responses to the RME but reported a low level of self-confidence (≤ 2/6) and when patients produced incorrect responses but maintained a high level of self-confidence (≥ 5/6).

### Preoperative neuropsychological assessment

Patients received a neuropsychological assessment, as part of their routine preoperative care. Scores were appropriately converted into Z-scores based on published French normative data (adjusted for educational level, age and sex). As some patients were not native French-speakers, they did not receive a full neuropsychological examination. Note that some neuropsychological tasks were tailored to the location of the tumor and, therefore, were not administered to all patients (e.g. the reading task was uniquely administered to right-handed patients with a left-sided tumor).

### Imaging acquisition and normalization

MRI acquisition (3 T, Siemens Avento, Siemens Medical Systems) was performed for all patients the day before surgery and 3 months after surgery, including Fluid-attenuated inversion recovery (FLAIR) sequences and T1-weighted 3D Gadolinium-enhanced sequences. The tumor volume was measured with a dedicated software (Myrian, Intrasense, Montpellier, France).

All 3-month postoperative MRI datasets were conformed to the standardized Montreal Neurological Institute (MNI) space, using SPM12 implemented in MATLAB environment (Release 2018a, The MathWorks Inc., Natick, NA, USA). All normalized MRIs were carefully checked to identify maladjusted deformations.

### Spatial topography of stimulations

Functional sites of interest were plotted in the MNI space using operative reports and intraoperative photographs. A similar method was previously described, and was reported to have a high level of reproducibility^7,44^. The MNI coordinates of each stimulation point were recorded. The spatial topography of the responsive cortical and subcortical stimulations was then plotted onto a three-dimensional MNI template using the MRIcroGL software (University of South Carolina, Columbia, USA).

### Disconnectome analysis

In order to define with matter pathways involved in self-evaluative processing, the disconnectome MAPS software was used, as part of the BCBToolkit^45^. For a given stimulation, Disconnectome MAPS provides a probability to be disconnected for every voxel of the MNI152 template, based on a tractography-based atlas of white matter^46^. A threshold of 0.00 was applied in the BCBToolKit command, meaning that all interindividual variation in the topological positioning of the tracts were considered. Given the spatial distribution of the responsive DES, individual disconnectome maps were overlaid in MRICroGL software with the third version of the automated anatomical labelling atlas (AAL3) and the Human Connectome Project (HCP) tractography database to extract voxels with maximal probability of disconnection (p_max_) and their corresponding MNI coordinates for each region of interest. An example of this methodology is detailed in Supplementary information—Methods. In addition, three average disconnectome maps depending on the location of the stimulations were established to report the maximal disconnectome overlapping (parietal and cingulum vs left fronto-temporo-insular vs right fronto-temporo-insular locations).

### Statistical analysis

Continuous variables were expressed as means ± standard deviation and categorical variables were expressed as numbers and proportions. Comparison of p_max_ within different white matter tracts were performed with the Kruskal–Wallis test. Post-hoc multiple pairwise comparisons were then performed with Dunn’s test. For all statistical analyses, adjusted P < 0.05 was considered statistically significant. Statistical tests were performed with Prism 7.0 software (GraphPad Software Inc., San Diego, CA, USA).

## Supplementary Information

Supplementary Information

## Abbreviations

AAL: Automated anatomical labelling atlas
AF: Arcuate fasciculus
DES: Direct electrostimulation
DO80: Dénomination orale à 80 items
HCP: Human connectome project
IFOF: Inferior fronto-occipital fasciculus
ILF: Inferior longitudinal fasciculus
MdLF: Middle longitudinal fasciculus
MNI: Montreal neurological institute
PPTT: Pyramid and palm tree task
RME: Read the mind in the eyes
SCI: Self-confidence index,
SLF: Superior longitudinal fasciculus
UF: Uncinate fasciculus
