# Supplementary material for: Disrupting self-evaluative processing with electrostimulation mapping during awake brain surgery
Source: Sci Rep. 2021 Apr 30;11:9386. doi: 10.1038/s41598-021-88916-y (PMC8087680; doi:10.1038/s41598-021-88916-y)
Supplement: Supplementary file 1 — Supplementary Information [file 41598_2021_88916_MOESM1_ESM.docx]

**Supplementary information**

**Disrupting self-evaluative processing with electrostimulation mapping during awake brain surgery**

Sam Ng, MD,*^1,2^ Guillaume Herbet, PhD,^1,2,3^ Anne-Laure Lemaitre, PhD,^1,2^ Sylvie Moritz-Gasser, PhD,^1,2,3^ and Hugues Duffau, MD, PhD^1,2,3^

^1^Department of Neurosurgery, Gui de Chauliac Hospital, Montpellier University Medical Center, Montpellier, France

^2^Institut de Génomique Fonctionnelle, Université de Montpellier, CNRS, INSERM, Montpellier, France

^3^Department of Speech-Language Pathology, University of Montpellier, Montpellier, France

**Corresponding author:**

*Sam Ng, MD

Department of Neurosurgery, Gui de Chauliac Hospital, Montpellier University Medical Center and INSERM U1191, Montpellier, France

80 Av Augustin Fliche, 34295 Montpellier, France

Phone: 33 4 67 33 66 12

Fax: 33 4 67 33 75 57

Email: [s-ng@chu-montpellier.fr](mailto:s-ng@chu-montpellier.fr)

**Supplementary information – Methods**


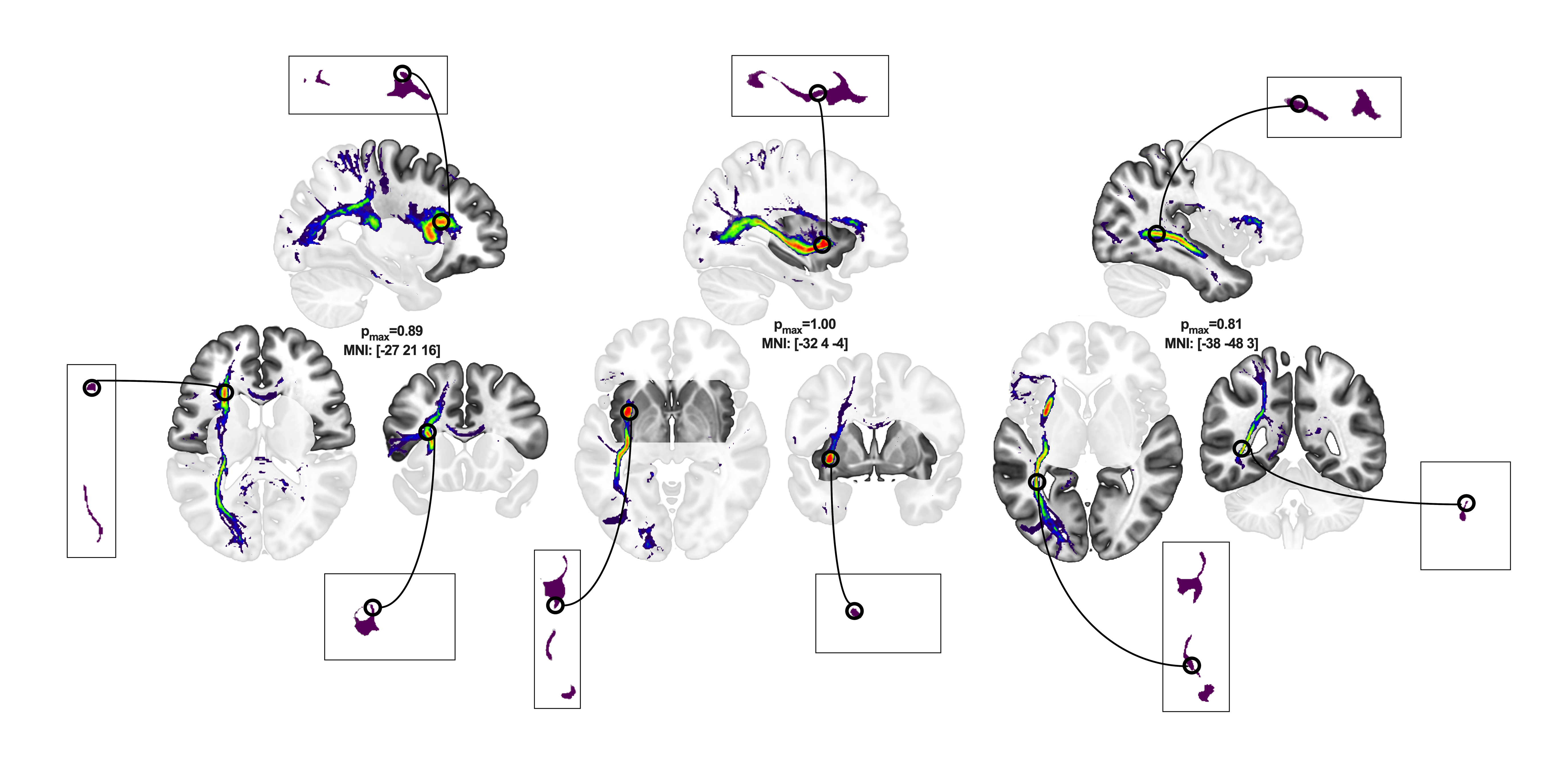


Supplementary figure

Example of determination of p_max_ within the left inferior fronto-occipital fasciculus (IFOF), including its frontal component (*left*), its insular component (*center*) and its occipital component (*right*). Templates of the IFOF, extracted from the Human Connectome Project (HCP) database are presented in purple.

Disconnectome analysis and determination of p_max_

In order to define withe matter pathways involved in self-evaluative processing, the disconnectome MAPS software was used, as part of the BCBToolkit. For a given stimulation, Disconnectome MAPS provides a probability to be disconnected for every voxel of the MNI152 template, based on a tractography-based atlas of white matter. A threshold of 0.00 was applied in the BCBToolKit command, meaning that all interindividual variation in the topological positioning of the tracts were considered.

Individual disconnectome maps were overlaid in MRICroGL software with templates of the different white matter tracts, extracted from the Human Connectome Project (HCP) tractography database.

Voxels with the maximal probability of disconnection (p_max_) and their corresponding MNI coordinates were extracted for each HCP white matter tracts.

Segmentation of the white matter tracts.

In order to define more precisely the p_max_ in different subcomponents of the main white matter tracts, individual disconnectome maps were overlaid with the HCP white matter tracts and with the “ch2better” template. This template is freely accessible when downloading the MRICro software at <https://www.mccauslandcenter.sc.edu/crnl/mricro>. The main white matter tracts segmentation was performed by anatomical segmentation, using the ch2better template (e.g. the p_max_ within the frontal component of the uncinate fascicle was determined exclusively within voxels belonging to the frontal lobe, whereas the p_max_ within the temporal component of the uncinate fascicle was determined exclusively within voxels belonging to the temporal lobe).

Segmentation of the inferior fronto-occipital fasciculus (IFOF).

The IFOF was segmented in three different components (see **supplementary figure**): a frontal component, an insular component and an occipital component.

Arbitrarily, the insular volume extracted from the third version of the automated anatomical labelling atlas (AAL3) was used to delineate insular edges in a reproducible manner (AAL3, template 33 and template 34).

Then, this template was overlaid with the HCP template of the IFOF and individual disconnectome maps. Portions of the IFOF located within the insula (according to AAL3 template) were eligible for the “insular component” (see the ***central part* of the supplementary figure**). Portions of the IFOF located anteriorly to the insula (according to AAL3 template) were eligible for the “frontal component” (see the ***left part* of the supplementary figure**). Portions of the IFOF located posteriorly to the insula (according to AAL3 template) were eligible for the “occipital component” (see the ***rightl part* of the supplementary figure**).

**Supplementary information - Results**

Individual P_max_ and related MNI coordinates (N=74)

| **Stimulation** | **IFOF1** | MNI | **IFOF2** | MNI | **IFOF3** | MNI | **ILF1** | MNI | **ILF2** | MNI | **UF1** | MNI | **UF2** | MNI | **MdLF1** | MNI | **MdlF2** | MNI | **SLF1** | MNI | **SLF2** | MNI | **AF1** | MNI | **AF2** | MNI | **Cing** | MNI |
| --- | --- | --- | --- | --- | --- | --- | --- | --- | --- | --- | --- | --- | --- | --- | --- | --- | --- | --- | --- | --- | --- | --- | --- | --- | --- | --- | --- | --- |
| 1 | 0,75 | -26; 35; -3 | 0,91 | -32;1;-9 | 1 | -41;-23;-12 | 1 | -43; -17; -16 | 1 | -43;-32;-13 | 0,76 | -23;24;-8 | 0,1 | -42; 6; -25 | 0,11 | -26; -48; 31 | 0 |  | 0 |  | 0 |  | 0,1 | -33; -4; 23 | 0,1 | -41; -37; 6 | 0 |  |
| 2 | 0,9 | 27; 30; 8 | 1 | 30; 14; -2 | 0,81 | 38; -27; -3 | 0,2 | 41; -16; -14 | 0,62 | 39; -50; 1 | 0 |  | 0,19 | 39; -5; -18 | 0,41 | 26; -47; 31 | 0,22 | 40; -25; 10 | 0,57 | 25; 39; 14 | 0,1 | 27; -41; 35 | 0,44 | 31; 32; 12 | 0,4 | 39; -50; 7 | 0 |  |
| 3 | 0,39 | -26; 36; -3 | 0,67 | -25; 8; -12 | 0,45 | -42; -39; -5 | 1 | -45; -20; -18 | 0,98 | -46; -27; -16 | 0,61 | -24; 11; -12 | 0,1 | -38; -5; -19 | 0,1 | -34; -33; 9 | 0 |  | 0,2 | -29; -1; 37 | 0,1 | -32; -47; 27 | 0,28 | -32; 11; 18 | 0,54 | -48; -35; -11 | 0 |  |
| 4 | 0,78 | -26; 20; 15 | 0,96 | -34; -13; -6 | 0,7 | -35; -41; -8 | 0,32 | -42; -20; -13 | 0,56 | -38; -44; 2 | 0,1 | -26; 18; -9 | 0,1 | -41; -4; -19 | 0,36 | -22; -50; 39 | 0,48 | -32; -34; 10 | 0,42 | -26; 21; 25 | 0 |  | 0,15 | -28; 16; 24 | 0,24 | -40; -41; 9 | 0 |  |
| 5 | 0,89 | -25; 16; 10 | 1 | -30; 10; 1 | 0,77 | -37; -30; -1 | 0,4 | -40; -14; -14 | 0,74 | -39; -33; -1 | 0 |  | 0,07 |  | 0,45 | -26; -46; 28 | 0,21 | -33; -31; 8 | 0,19 | -27; 24; 22 | 0,1 | -26; -37; 37 | 0,19 | -29; 19; 23 | 0,27 | -41; -37; 6 | 0 |  |
| 6 | 1 | -26; 22; 12 | 0,82 | -34; -5; -7 | 0,78 | -36; -32; 2 | 0 |  | 0,73 | -39; -41; 1 | 0,03 |  | 0,03 |  | 0 |  | 0 |  | 0 |  | 0 |  | 0,7 | -32; -2; 18 | 0 |  | 0 |  |
| 7 | 0,89 | -26; 35; -4 | 1 | -33; -2; -9 | 1 | -42; -29; -11 | 1 | -41; -20; -13 | 0,95 | -41; -31; -10 | 0,87 | -21; 22; -10 | 0 |  | 0 |  | 0 |  | 0,03 |  | 0 |  | 0 |  | 0 |  | 0 |  |
| 8 | 1 | 27; 35; -2 | 1 | 33; -2; -11 | 1 | 40; -22; -11 | 0,96 | 42; -21; -14 | 0,81 | 41; -44; -8 | 0,95 | 27; 14; -9 | 0,81 | 39; -8; -19 | 0,17 | 23; -19; 40 | 0,19 | 34; -33; 12 | 0,45 | 25; 39; 12 | 0,1 | 30; -41; 31 | 0,53 | 29; 37; 6 | 0,12 | 39; -48; 10 | 0 |  |
| 9 | 1 | 29; 35; 3 | 1 | 33; 9; -8 | 0,96 | 39; -25; -7 | 0,87 | 41; -22; -11 | 0,78 | 40; -47; -3 | 0,59 | 30; 12; -10 | 0,3 | 40; -9; -17 | 0,26 | 25; -48; 34 | 0,34 | 36; -31; 9 | 0,41 | 26; 39; 10 | 0,15 | 26; -36; 38 | 0,24 | 31; 35; 9 | 0,68 | 42; -49; -2 | 0 |  |
| 10 | 0,96 | -25; 18; 4 | 1 | -32; 3; -7 | 0,98 | -40; -31; -3 | 0,75 | -41; -23; -10 | 0,77 | -37; -47; 3 | 0,03 |  | 0,01 |  | 0 |  | 0 |  | 0,5 | -26; 32; 16 | 0 |  | 0 |  | 0 |  | 0 |  |
| 11 | 0,87 | -27; 35; -1 | 0,89 | -34; -7; -11 | 0,89 | -42; -31; -9 | 0,63 | -43; -12; -18 | 0,88 | -43; -35; -9 | 0,5 | -24; 17; -9 | 0 |  | 0 |  | 0 |  | 0 |  | 0 |  | 0 |  | 0 |  | 0 |  |
| 12 | 0,85 | 28; 36; 1 | 1 | 35; -2; -9 | 0,9 | 40; -29; -6 | 0,99 | 41; -22; -12 | 0,82 | 39; -51; -3 | 0,2 | 27; 14; -10 | 0,5 | 39; -10; -16 | 0 |  | 0 |  | 0,34 | 27; 43; 4 | 0,1 | 30; -44; 31 | 0,18 | 30; 18; 20 | 0,76 | 42; -47; -2 | 0 |  |
| 13 | 0,85 | 27; 34; -2 | 1 | 34; 6; -7 | 0,99 | 39; -26; -6 | 0,73 | 41; -12; -17 | 0,89 | 32; -64; 0 | 0,74 | 25; 20; -10 | 0,93 | 38; -8; -15 | 0,1 | 25; -41; 40 | 0,19 | 36; -32; 9 | 0,24 | 25; 40; 11 | 0,1 | 29; -41; 33 | 0,22 | 32; 31; 14 | 0,86 | 41; -48; -3 | 0 |  |
| 14 | 0,99 | -20; 22; -10 | 1 | -27; 9; -11 | 0,5 | -43; -30; -11 | 0,5 | -40; -16; -19 | 0,69 | -26; -68; -1 | 1 | -23; 13; -13 | 0,61 | -36; -6; -19 | 0 |  | 0 |  | 0,01 |  | 0 |  | 0,02 |  | 0 |  | 0 |  |
| 15 | 0,7 | 27; 31; 4 | 0,99 | 36; -7; -9 | 1 | 39; -27; -3 | 0,8 | 44; -17; -14 | 0,79 | 25; -80; 0 | 0,14 | 29; 14; -10 | 0,16 | 39; -6; -14 | 0,11 | 27; -50; 31 | 0,18 | 41; -26; 3 | 0,25 | 27; 39; 9 | 0 |  | 0,13 | 30; 35; 8 | 0,42 | 40; -49; 8 | 0 |  |
| 16 | 0,74 | -24; 38; -5 | 1 | -32; -2; -11 | 0,82 | -41; -21; -14 | 0,83 | -42; -19; -16 | 0,64 | -25; -75; -1 | 0,93 | -26; 10; -11 | 0,63 | -36; -4; -19 | 0 |  | 0 |  | 0 |  | 0 |  | 0 |  | 0 |  | 0 |  |
| 17 | 0,5 | -26; 20; 15 | 0,95 | -29; 12; 4 | 0,73 | -33; -45; 14 | 0 |  | 0,62 | -34; -42; 12 | 0 |  | 0,08 |  | 0 |  | 0 |  | 0 |  | 0 |  | 0 |  | 0 |  | 0 |  |
| 18 | 0,1 | -25; 30; -3 | 0,77 | -33; 0; -12 | 0,12 | -35; -19; -4 | 0,59 | -43; 8; -28 | 0,1 | -38; -45; 1 | 0,8 | -24; 9; -14 | 0,82 | -38; -4; -23 | 0,13 | -24; -43; 37 | 0 |  | 0,02 |  | 0 |  | 0 |  | 0,03 |  | 0 |  |
| 19 | 0 |  | 0 |  | 0 |  | 0 |  | 0 |  | 0 |  | 0 |  | 0 |  | 0 |  | 0 |  | 0 |  | 0 |  | 0 |  | 1 | 10; -38; 30 |
| 20 | 0,32 | -26; 35; -4 | 0,86 | -29; 2; -11 | 0,13 | -37; -38; 5 | 0,65 | -38; -10; -22 | 0,18 | -19; -77; -3 | 0,84 | -23; 11; -14 | 0,88 | -37; -6; -20 | 0,06 |  | 0 |  | 0,9 |  | 0 |  | 0,1 | -29; 13; 32 | 0 |  | 0 |  |
| 21 | 0,99 | 24; 33; -7 | 1 | 26; 13; -10 | 0,7 | 39; -11; -16 | 0,68 | 41; -16; -16 | 0,7 | 40; -11; -19 | 1 | 26; 14; -10 | 0,76 | 39; -9; -19 | 0,03 |  | 0 |  | 0 |  | 0 |  | 0 |  | 0 |  | 0 |  |
| 22 | 0,29 | 26; 28; 4 | 0,73 | 33; -5; -7 | 0,34 | 36; -30; 2 | 0,1 | 41; -25; -11 | 0,36 | 35; -58; 9 | 0 |  | 0,04 |  | 0,14 | 28; -45; 29 | 0,17 | 41; -29; 6 | 0,46 | 27; 26; 22 | 0 |  | 0,5 | 29; 23; 20 | 0,1 | 44; -47; -1 | 0 |  |
| 23 | 0,49 | -18; 20; -12 | 0,76 | -31; 4; -11 | 0,14 | -37; -38; 3 | 0,8 | -40; -5; -25 | 0,18 | -18; -84; -4 | 0,83 | -23; 11; -15 | 0,93 | -39; -3; -24 | 0,03 |  | 0 |  | 0 |  | 0 |  | 0,15 | -49; -25; -18 | 0 | - | 0 |  |
| 24 | 0,52 | 27; 32; 8 | 0,85 | 33; 1; -6 | 0,59 | 32; -37; 14 | 0,15 | 42; 1; -28 | 0,42 | 37; -51; 9 | 0 |  | 0 |  | 0,47 | 24; -49; 37 | 0,48 | 49; -18; 3 | 0,22 | 27; 10; 36 | 0 |  | 0,47 | 29; 16; 29 | 0,67 | 43; -49; -2 | 0 |  |
| 25 | 0,79 | 23; 32; -8 | 1 | 33; 4; -10 | 0,98 | 40; -15; -13 | 0,88 | 41; -13; -19 | 0,66 | 43; -30; -13 | 0,82 | 27; -14; -10 | 1 | 37; -3; -16 | 0 |  | 0,1 | 35; -33; 11 | 0,17 | 25; 36; 17 | 0,1 | 34; -50; 28 | 0,2 | 31; 32; 12 | 0,6 | 43; -47; -4 | 0 |  |
| 26 | 0,8 | 27; 33; 6 | 1 | 34; 3; -7 | 0,99 | 38; -26; -5 | 0,29 | 41; -19; -13 | 0,78 | 33; -64; 1 | 0,18 | 25; 20; -10 | 0 |  | 0,63 | 27; -50; 28 | 0,32 | 32; -30; 13 | 0,38 | 25; 37; 17 | 0,1 | 33; -50; 28 | 0,34 | 30; 34; 10 | 0,54 | 41; -49; 3 | 0,1 | 17; 37; 15 |
| 27 | 0,12 | 27; 33; 9 | 0,11 | 35; 0; -6 | 0,12 | 33; -22; 1 | 0 |  | 0 |  | 0 |  | 0,1 | 40; -7; -17 | 0,19 | 24; -48; 38 | 0 |  | 0 |  | 0 |  | 0,13 | 33; 32; 12 | 0 |  | 0 |  |
| 28 | 0,73 | -26; 28; 12 | 1 | -34; 3; -5 | 0,93 | -39; -36; -1 | 0,6 | -40; -23; -8 | 0,79 | -39; -45; 1 | 0,05 |  | 0 |  | 0,41 | -21; -51; 39 | 0,1 | -32; -40; 17 | 0,35 | -27; 18; 24 | 0 |  | 0,15 | -29; 18; 25 | 0,1 | -48; -35; -12 | 0 |  |
| 29 | 0,69 | -26; 35; -3 | 0,87 | -33; -2; -9 | 0,99 | -40; -44; 1 | 0,75 | -40; -22; -11 | 0,99 | -42; -44; -2 | 0,76 | -23; 23; -7 | 0 |  | 0,1 | -26; -50; 29 | 0 | - | 0,33 | -32; 26; 19 | 0,1 | -38; -39; 31 | 0,8 | -34; -7; 24 | 0,97 | -46; -44; -8 | 0 |  |
| 30 | 0,66 | -16; 34; -15 | 0,92 | -32; -1; -12 | 0,16 | -34; -53; 7 | 0,76 | -40; -12; -19 | 0,16 | -23; -75; -2 | 0,82 | -22; 11; -14 | 1 | -36; -6; -19 | 0,05 |  | 0 |  | 0,1 |  | 0 |  | 0 |  | 0,11 | -50; -26; -18 | 0 |  |
| 31 | 0,53 | -18; 33; -13 | 0,97 | -32; -1; -11 | 0,39 | -42; -19; -15 | 0,78 | -40; -13; -19 | 0,43 | -34; -47; -6 | 0,89 | -23; 11; -13 | 0,53 | -40; 7; -31 | 0 |  | 0 |  | 0,14 | -28; 7; 37 | 0 |  | 0 |  | 0,23 | -48; -47; -16 | 0 |  |
| 32 | 0,96 | -27; 37; -3 | 1 | -32; 3; -11 | 0,65 | -42; -26; -12 | 0,79 | -41; -18; -16 | 0,63 | -43; -37; -9 | 0,93 | -23; 21; -10 | 0,76 | -40; 3; -26 | 0 |  | 0 |  | 0,13 | -29; 41; 6 | 0 |  | 0,1 | -33; -14; 25 | 0,39 | -48; -27; -16 | 0 |  |
| 33 | 0 |  | 0,13 | 27; 21; 1 | 0,1 | 36; -38; 9 | 0 |  | 0 |  | 0,01 |  | 0 |  | 0 |  | 0,37 | 32; -34; 15 | 0 |  | 0 |  | 0 |  | 0 |  | 0 |  |
| 34 | 0,98 | 19; 27; -11 | 1 | 32; 6; -10 | 0,74 | 41; -17; -14 | 0,84 | 41; -12; -19 | 0,76 | 36; -55; -4 | 1 | 22; 15; -15 | 0,98 | 37; -4; -19 | 0 |  | 0 |  | 0,1 | 27; 34; 15 | 0 |  | 0,1 | 33; 34; 11 | 0,51 | 41; -50; -3 | 0 |  |
| 35 | 0,85 | 23; 34; -8 | 1 | 34; 0; -12 | 0,8 | 42; -23; -13 | 0,89 | 41; -14; -18 | 0,78 | 36; -53; -4 | 0,88 | 25; 14; -13 | 0,93 | 39; -5; -21 | 0 |  | 0 |  | 0,19 | 25; 37; 16 | 0,1 | 31; -44; 31 | 0,15 | 30; 38; 4 | 0,32 | 41; -50; -2 | 0 |  |
| 36 | 0,74 | -26; 25; 12 | 0,99 | -34; -5; -7 | 0,93 | -39; -29; -4 | 0,34 | -41; -22; -10 | 0,82 | -38; -45; 3 | 0,05 |  | 0 |  | 0,57 | -23; -44; 31 | 0,5 | -32; -33; 9 | 0,45 | -26; 21; 24 | 0,22 | -26; -35; 37 | 0,25 | -29; 18; 22 | 0,1 | -48; -32; -15 | 0 |  |
| 37 | 0,75 | -31; 22; 16 | 0,46 | -26; 18; 7 | 0,6 | -39; -45; 4 | 0,41 | -45; -13; -24 | 0,75 | -44; -39; -6 | 0,1 | -25; 25; -8 | 0,1 | -40; 4; -28 | 0 |  | 0,6 | -37; -33; 7 | 0 |  | 0,5 | -29; -23; 40 | 0,87 | -31; 11; 18 | 0,71 | -47; -39; -9 | 0 |  |
| 38 | 0,93 | 27; 32; 1 | 1 | 33; 6; -6 | 1 | 40; -23; -10 | 0,71 | 41; -13; -14 | 0,86 | 34; -62; 0 | 0,7 | 27; 18; -9 | 0,59 | 39; -8; -16 | 0,21 | 25; -48; 37 | 0,19 | 37; -33; 9 | 0,41 | 27; 38; 10 | 0,1 | 33; -49; 29 | 0,36 | 32; 35; 6 | 0,35 | 39; -50; 9 | 0 |  |
| 39 | 0,81 | -24; 29; -7 | 1 | -34; -6; -14 | 0,66 | -43; -22; -15 | 0,74 | -40; -13; -19 | 0,43 | -26; -68; -1 | 0,81 | -25; 14; -12 | 0,81 | -37; -9; -17 | 0 |  | 0 |  | 0,6 |  | 0 |  | 0 |  | 0,25 | -48; -27; -16 | 0 |  |

| **Stimulation** | **IFOF1** | MNI | **IFOF2** | MNI | **IFOF3** | MNI | **ILF1** | MNI | **ILF2** | MNI | **UF1** | MNI | **UF2** | MNI | **MdLF1** | MNI | **MdlF2** | MNI | **SLF1** | MNI | **SLF2** | MNI | **AF1** | MNI | **AF2** | MNI | **Cing** | MNI |
| --- | --- | --- | --- | --- | --- | --- | --- | --- | --- | --- | --- | --- | --- | --- | --- | --- | --- | --- | --- | --- | --- | --- | --- | --- | --- | --- | --- | --- |
| 40 | 0,78 | 27; 34; 6 | 1 | 34; -7; -9 | 1 | 39; -33; -4 | 0 |  | 0,8 | 33; -59; 1 | 0,1 | 23; 10; -12 | 0,1 | 38; -3; -19 | 0,04 |  | 0,04 |  | 0,41 | 26; 37; 12 | 0 |  | 0,17 | 32; 35; 6 | 0,43 | 40; -50; 4 | 0 |  |
| 41 | 0,1 | 30; 34; 3 | 0 |  | 0 |  | 0 |  | 0 |  | 0,03 |  | 0 |  | 0,06 |  | 0 |  | 0 |  | 0 |  | 0,1 | 33; 30; 15 | 0 |  | 0,1 | 11; -4; 34 |
| 42 | 0 |  | 0,17 | 33; -3; -8 | 0,21 | 36; -35; 7 | 0 |  | 0,1 | 35; -54; 14 | 0 |  | 0 |  | 0 |  | 0 |  | 0,51 | 28; 23; 25 | 0 |  | 0,62 | 29; 21; 23 | 0,1 | 44; -50; -3 | 0 |  |
| 43 | 0,72 | 25; 26; 3 | 0,99 | 33; -4; -9 | 1 | 39; -30; -4 | 0,59 | 44; -13; -16 | 0,84 | 36; -60; 1 | 0,17 | 25; 19; -11 | 0,17 | 39; -5; -16 | 0,02 |  | 0 |  | 0,32 | 24; 45; 10 | 0 |  | 0,19 | 30; 34; 10 | 0,75 | 41; -48; 1 | 0 |  |
| 44 | 1 | -26; 24; 15 | 0,94 | -24; 21; 11 | 0,77 | -38; -36; 1 | 0,28 | -40; -14; -15 | 0,73 | -37; -50; 4 | 0 |  | 0,07 |  | 0,6 | -25; -45; 29 | 0,37 | -34; -32; 6 | 0,48 | -28; 27; 20 | 0,18 | -26; -37; 37 | 0,57 | -29; 23; 20 | 0,24 | -47; -50; -6 | 0 |  |
| 45 | 1 | -25; 32; 8 | 0,86 | -32; 4; -6 | 0,89 | -38; -29; -4 | 0,32 | -42; -18; -16 | 0,62 | -40; -33; -2 | 0,1 | -29; 12; -8 | 0,26 | -37; -8; -17 | 0,18 | -26; -49; 28 | 0,29 | -32; -34; 10 | 0,26 | -26; 15; 30 | 0 |  | 0,13 | -31; 11; 20 | 0,19 | -42; -48; 0 | 0 |  |
| 46 | 0,63 | -26; 22; 12 | 0,82 | -34; -9; -7 | 0,68 | -35; -32; 4 | 0,3 | -41; -16; -13 | 0,58 | -36; -43; 8 | 0 |  | 0,06 |  | 0,45 | -25; -42; 29 | 0,28 | -43; -30; 6 | 0,32 | -25; 19; 29 | 0,1 | -26; -37; 36 | 0,41 | -29; 18; 23 | 0,5 | -42; -41; 0 | 0,1 | -12; -14; 34` |
| 47 | 0,1 | 27; 32; 7 | 0,11 | 34; 3; -7 | 0,1 | 36; -34; 4 | 0 |  | 0,1 | 32; -69; 9 | 0,01 |  | 0 |  | 0,08 |  | 0 |  | 0,1 | 28; 37; 12 | 0 |  | 0,35 | 32; 37; 9 | 0 |  | 0 |  |
| 48 | 0 |  | 0 |  | 0 |  | 0 |  | 0 |  | 0 |  | 0 |  | 0 |  | 0 |  | 0 |  | 0 |  | 0 |  | 0 |  | 0,49 | 13; 25; 32 |
| 49 | 0,28 | -25; 19; 12 | 0,42 | -31; 0; -7 | 0 |  | 0 |  | 0 |  | 0 |  | 0 |  | 0,68 | -21; -52; 41 | 0,14 | -37; -27; 2 | 0 |  | 0 |  | 0 |  | 0 |  | 0,85 | -18; -49; 37 |
| 50 | 0,59 | -16; 30; -14 | 1 | -32; 0; -12 | 0,1 | -36; -47; 6 | 0,76 | -40; -11; -20 | 0,16 | -23; -73; -2 | 0,89 | -22; 12; -15 | 0,92 | -34; -4; -16 | 0,09 | -26; -47; 31 | 0 |  | 0,1 | -27; 9; 35 | 0 |  | 0 |  | 0,24 | -48; -28; -15 | 0 |  |
| 51 | 0,52 | -26; 35; -4 | 0,72 | -27; 4; -11 | 0,75 | -43; -26; -13 | 0,82 | -42; -13; -22 | 1 | -44; -33; -13 | 0,55 | -21; 20; -11 | 0,3 | -37; -9; -17 | 0,07 | -25; -50; 31 | 0,04 |  | 0,13 | -32; 32; 12 | 0 |  | 0 |  | 0,25 | -33; 10; 17 | 0 |  |
| 52 | 0 |  | 0 |  | 0 |  | 0 |  | 0 |  | 0 |  | 0 |  | 0 |  | 0 |  | 0 |  | 0 |  | 0 |  | 0 |  | 1 | -18; -41; 34 |
| 53 | 0,86 | -26; 34; 6 | 0,86 | -32; 4; -6 | 0,87 | -37; -29; -3 | 0,35 | -41; -22; -11 | 0,71 | -40; -35; -4 | 0,2 | -31; 13; -5 | 0,25 | -40; -10; -18 | 0,2 | -26; -52; 28 | 0,1 | -34; -30; 6 | 0,99 | -26; 38; 12 | 0,12 | -29; -52; 29 | 0,1 | -34; -2; 23 | 0,1 | -48; -24; -16 | 0,12 | -9; -41; 26 |
| 54 | 0,95 | -28; 21; 16 | 0,89 | -29; 12; 4 | 0,74 | -36; -33; 3 | 0,29 | -40; -14; -15 | 0,53 | -34; -53; 10 | 0,1 | -29; 26; -11 | 0,16 | -39; -10; -16 | 0,32 | -25; -45; 31 | 0,12 | -37; -32; 6 | 0,46 | -27; 19; 23 | 0,19 | -25; -38; 77 | 0,11 | -29; 19; 23 | 0,36 | -42; -37; 6 | 0 |  |
| 55 | 0,41 | 28; 32; 5 | 0,1 | 34; 15; -6 | 0,1 | 39; -37; 3 | 0 |  | 0,16 | 34; -67; 9 | 0 |  | 0 |  | 0 |  | 0 |  | 0 |  | 0 |  | 0,06 |  | 0 |  | 0 |  |
| 56 | 0,3 | -27; 23; 16 | 0,89 | -34; -13; -5 | 0,79 | -33; -24; -2 | 0,32 | -41; -19; -13 | 0,6 | -37; -33; 1 | 0 |  | 0 |  | 0,17 | -23; -44; 42 | 0,13 | -34; -32; 11 | 0,5 | -26; 20; 25 | 0,19 | -27; -35; 35 | 0,5 | -29; 18; 22 | 0,16 | -37; -38; 15 | 0,12 | -17; -51; 21 |
| 57 | 0,41 | 27; 32; 5 | 0,32 | 34; 5; -5 | 0,48 | 32; -49; 22 | 0,1 | 44; -19; -14 | 0,21 | 35; -61; 12 | 0 |  | 0,02 |  | 0,26 | 22; -47; 47 | 0,12 | 39; -24; 9 | 0,5 | 27; 26; 23 | 0 |  | 0,65 | 33; 25; 18 | 0,31 | 46; -47; -2 | 0 |  |
| 58 | 0,45 | 26; 29; 8 | 0,81 | 34; -12; -5 | 0,62 | 37; -33; 3 | 0,18 | 43; -16; -16 | 0,6 | 34; -58; 15 | 0 |  | 0,11 | 41; -6; -19 | 0,25 | 24; -45; 37 | 0,38 | 36; -34; 12 | 0,33 | 25; 36; 17 | 0,1 | 26; -32; 39 | 0,79 | 29; 30; 14 | 0,37 | 38; -51; 7 | 0 |  |
| 59 | 0,9 | -26; 35; -4 | 1 | -34; -7; -13 | 0,84 | -43; -26; -12 | 0,88 | -40; -16; -16 | 0,88 | -42; -33; -9 | 0,84 | -26; 14; -11 | 1 | -35; -5; -14 | 0,1 | -25; -47; 31 | 0 |  | 0,22 | -27; 37; 10 | 0 |  | 0,1 | -28; 14; 28 | 0,49 | -48; -27; -15 | 0 |  |
| 60 | 0 |  | 0 |  | 0 |  | 0 |  | 0 |  | 0 |  | 0 |  | 0 |  | 0 |  | 0 |  | 0 |  | 0 |  | 0 |  | 0,33 | -8; 4; 37 |
| 61 | 0 |  | 0 |  | 0 |  | 0 |  | 0 |  | 0 |  | 0 |  | 0 |  | 0 |  | 0 |  | 0 |  | 0 |  | 0 |  | 0,32 | -12; -20; 35 |
| 62 | 0 |  | 0 |  | 0 |  | 0 |  | 0 |  | 0 |  | 0 |  | 0 |  | 0 |  | 0 |  | 0 |  | 0 |  | 0 |  | 0,29 | -14; -46; 29 |
| 63 | 0,77 | -27; 36; -1 | 0,87 | -33; -10; -13 | 0,89 | -41; -39; -2 | 0,84 | -43; -22; -13 | 1 | -43; -36; -8 | 0,65 | -26; 9; -11 | 0,1 | -38; -7; -19 | 0 |  | 0 |  | 0,5 | -34; 34; 16 | 0 |  | 0,83 | -32; 9; 19 | 0,99 | -47; -38; -11 | 0 |  |
| 64 | 0,1 | 27; 37; 5 | 0,36 | 32; -6; -8 | 0,14 | 36; -35; 6 | 0 |  | 0,18 | 35; -58; 9 | 0 |  | 0 |  | 0,1 | 23; -50; 45 | 0,1 | 36; -33; 10 | 0,75 | 32; 23; 22 | 0 |  | 0,99 | 31; 23; 20 | 0,23 | 46; -47; -1 | 0 |  |
| 65 | 0,89 | -26; 35; -4 | 1 | -33; -6; -11 | 0,99 | -43; -30; -11 | 1 | -41; -18; -16 | 0,96 | -43; -33; -12 | 0,87 | -25; 12; -11 | 1 | -35; -6; -14 | 0 |  | 0 |  | 0,12 | -26; 23; 23 | 0,1 | -27; -44; 33 | 0 |  | 0,25 | -47; -31; -11 | 0 |  |
| 66 | 0,8 | 27; 32; 5 | 1 | 35; -8; -7 | 0,91 | 39; -31; -3 | 0 |  | 0,77 | 39; -51; 1 | 0 |  | 0,19 | 37; -5; -11 | 0,27 | 24; -49; 38 | 0,4 | 37; -30; 6 | 0,32 | 24; 38; 17 | 0,1 | 32; -48; 31 | 0,27 | 32; 32; 12 | 0,4 | 41; -48; 2 | 0 |  |
| 67 | 0,77 | -33; 22; 15 | 0,51 | -33; 0; -8 | 0,86 | -43; -35; -7 | 0,59 | -43; -22; -12 | 1 | -42; -41; -5 | 0 |  | 0 |  | 0 |  | 0,08 |  | 0,7 |  | 0 |  | 0,88 | -32; 2; 20 | 0,98 | -46; -37; -8 | 0 |  |
| 68 | 0 |  | 0 |  | 0,39 | -36; -51; 9 | 0,19 | -38; -5; -30 | 0,46 | -35; -53; 12 | 0 |  | 0 |  | 0 |  | 0 |  | 1 | -29; -2; 34 | 0 |  | 0,95 | -32; -1; 31 | 0,31 | -40; -50; 4 | 0 |  |
| 69 | 0,83 | -27; 35; 0 | 0,87 | -33; -7; -12 | 1 | -43; -24; -12 | 1 | -43; -19; -14 | 0,98 | -44; -37; -9 | 0,74 | -23; 23; -6 | 0,1 | -37; -7; -16 | 0 |  | 0 |  | 0,11 | -26; 9; 39 | 0,1 | -35; -62; 31 | 0,28 | -32; 4; 18 | 0,26 | -46; -38; -8 | 0 |  |
| 70 | 0,89 | -26; 35; -4 | 1 | -34; 1; -9 | 0,87 | -41; -20; -14 | 0,88 | -40; -16; -16 | 0,72 | -43; -29; -14 | 0,86 | -26; 11; -12 | 1 | -34; -4; -14 | 0 |  | 0 |  | 0,1 | -29; 32; 15 | 0 |  | 0,1 | -27; 11; 28 | 0,38 | -47; -33; -13 | 0 |  |
| 71 | 0,9 | -26; 34; -4 | 1 | -33; 1; -10 | 0,67 | -41; -26; -10 | 0,83 | -39; -12; -16 | 0,62 | -43; -39; -8 | 0,79 | -25; 15; -12 | 0,77 | -41; 3; -26 | 0,02 |  | 0 |  | 0,19 | -27; 35; 12 | 0 |  | 0,1 | -28; 12; 26 | 0,47 | -48; -26; -15 | 0 |  |
| 72 | 0,94 | -32; 21; 15 | 0,32 | -27; 14; 6 | 0,7 | -39; -46; 3 | 0,14 | -43; -10; -25 | 0,7 | -39; -46; 3 | 0 |  | 0,09 |  | 0 |  | 0,23 | -40; -31; 6 | 0,23 | -29; 21; 22 | 0 |  | 0,54 | -32; 15; 20 | 0,58 | -51; -29; -15 | 0 |  |
| 73 | 0 |  | 0,1 | -33. 6; -5 | 0,53 | -37; -50; 7 | 0 |  | 0,56 | -38; -50; 6 | 0 |  | 0 |  | 0 |  | 0 |  | 0,71 | -26; 7; 34 | 0 |  | 0,99 | -30; 6; 26 | 0,4 | -41; -51; 2 | 0 |  |
| 74 | 0,1 | -31; 21; 16 | 0,78 | -34; -9; -7 | 0,6 | -36; -34; 4 | 0,27 | -41; -20; -12 | 0,53 | -33; -45; 15 | 0 |  | 0 |  | 0,36 | -26; -41; 27 | 0,1 | -43; -26; 6 | 0,37 |  | 0 |  | 0,27 | -31; 1; 17 | 0,14 | -51; -28; -14 | 0 |  |

AF1: Arcuate fasciculus (frontal component), AF2: Arcuate fasciculus (temporal component), Cing: Cingulum, ILF1: Inferior longitudinal fasciculus (temporal component), ILF2: inferior longitudinal fasciculus (occipital component), IFOF1: inferior fronto-occipital fasciculus (frontal component), IFOF2: inferior fronto-occipital fasciculus (insular component), IFOF3: inferior fronto-occipital fasciculus (occipital component), MdLF1: Middle longitudinal fasciculus (parietal component), MdLF2: Middle longitudinal fasciculus (temporal component), MNI: Montreal Neurological Institute, SLF1: Superior longitudinal fasciculus (frontal component), SLF2: Superior longitudinal fasciculus (parietal component), UF1: Uncinate fasciculus (frontal component), UF2: Uncinate fasciculus (temporal component).

Note that MNI coordinates were not reported in case of P_max_<0.10. Note that the same value of P_max_ may have been reached in different locations, and that only one location was arbitrarily reported.
